# Effects of Pneumoperitoneum on Mean Arterial Pressure and Hypotension Prediction Index Responses During Hemorrhage and Transfusion in a Porcine Model

**DOI:** 10.3390/jcm15218509

**Published:** 2026-11-02

**Authors:** Woo-Jin Kim, Dong-Kyu Kim, Yerin Yoon, Hyun-Seok Kim, Woo-Young Seo, Sung-Hoon Kim

**Affiliations:** 1 Department of Anesthesiology and Pain Medicine, Asan Medical Center, Brain Korea 21 Project, University of Ulsan College of Medicine, 88, Olympic-Ro 43-Gil, Seoul 05505, Republic of Korea; wj888.kim@gmail.com (W.-J.K.); yr0780@gmail.com (Y.Y.); zephyrus02@gmail.com (W.-Y.S.); 2 Department of Biomedical Engineering, Asan Institute of Life Sciences, University of Ulsan College of Medicine, Seoul 05505, Republic of Korea; dien.eaststar@gmail.com; 3 Big Data Research Center, Asan Institute for Life Sciences, Asan Medical Center, Seoul 05505, Republic of Korea; khs0330kr@gmail.com

**Keywords:** pneumoperitoneum, mean arterial pressure, hypotension prediction index, hemorrhage, blood transfusion, swine

## Abstract

**Background/Objectives:** CO_2_ pneumoperitoneum (PnP) alters hemodynamics during laparoscopic surgery. We examined whether PnP modifies mean arterial pressure (MAP) and hypotension prediction index (HPI) responses to controlled, stepwise hemorrhage and retransfusion in a porcine model. **Methods:** This exploratory secondary analysis included recordings from three PnP-only pilot animals and nine animals with analyzable data from a randomized crossover experiment involving both PnP and No-PnP conditions. Blood was withdrawn and retransfused in 100 mL increments up to 600 mL per phase under both PnP (15 mmHg) and No-PnP conditions. MAP and HPI were averaged within protocol windows and analyzed using linear mixed-effects models to assess changes across protocol windows, condition-by-window interactions, and paired within-animal differences. **Results:** Twenty-one hemorrhage–transfusion sets from 12 animals were analyzed, comprising 12 PnP and nine No-PnP sets. MAP decreased and HPI increased overall during hemorrhage, with the opposite pattern observed during retransfusion in both conditions (all protocol-window effects, *p* < 0.001). No significant condition-by-window interactions were detected during hemorrhage (MAP, *p* = 0.407; HPI, *p* = 0.925) or retransfusion (MAP, *p* = 0.999; HPI, *p* = 0.910). Within-animal differences did not vary significantly across protocol windows in either phase. **Conclusions:** No significant PnP-related differences in the patterns of MAP and HPI change were detected in this porcine model. These findings provide experimental context for interpreting MAP and HPI during blood loss in laparoscopic surgery; clinical studies are needed to determine whether HPI adds predictive information beyond MAP.

## 1. Introduction

Perioperative hemodynamic management aims to maintain adequate arterial pressure and organ perfusion during anesthesia and surgery [1,2]. The severity and duration of intraoperative hypotension have been associated with acute kidney injury, myocardial injury, and adverse cardiovascular and cerebrovascular outcomes after noncardiac surgery [3,4,5]. Mean arterial pressure (MAP) is routinely monitored to detect hypotension, whereas the hypotension prediction index (HPI) is an arterial waveform-derived score intended to predict impending hypotension [6]. Trials of HPI-guided management have reported differing effects on intraoperative hypotension, indicating that its clinical effects may depend on the monitoring and treatment strategy and surgical setting [7,8].

During laparoscopic surgery, CO_2_ pneumoperitoneum (PnP) alters cardiopulmonary interactions and can increase MAP and systemic vascular resistance while reducing cardiac output [9]. Changes in ventricular loading, surgical positioning, and neurohumoral responses may contribute to these effects [10,11]. Consequently, changes in arterial pressure during PnP may not directly reflect changes in circulating blood volume, complicating interpretation during acute blood loss.

HPI-guided management has been investigated during robot-assisted radical prostatectomy and radical cystectomy, including both laparoscopic and robotic procedures [12,13]. However, these studies did not isolate PnP-related changes in HPI responses during controlled blood-volume perturbations. Studies comparing HPI with MAP have reported similar predictive performance and a strong correlation between the two variables [14,15], supporting their combined evaluation during PnP.

In porcine models, HPI increased during graded hemorrhage accompanied by impaired intestinal microcirculation [16] and during esmolol-induced negative inotropy [17]. These findings indicate that HPI responses are not specific to blood-volume depletion. However, whether PnP modifies the patterns of MAP and HPI change during standardized hemorrhage and subsequent blood replacement remains unclear.

We therefore performed an exploratory secondary analysis of prospectively collected recordings from a controlled porcine hemorrhage–transfusion experiment. We aimed to characterize MAP and HPI responses during stepwise hemorrhage and retransfusion under PnP and No-PnP conditions, assess whether their patterns of change differed between conditions, and determine whether within-animal PnP–No-PnP differences varied across protocol windows within each phase.

## 2. Materials and Methods

### 2.1. Study Overview

The present study was an exploratory secondary analysis of prospectively collected MAP and HPI recordings from a porcine hemorrhage–transfusion experiment. The parent experiment was designed to compare the responses of pulse pressure variation, stroke volume variation, and heart sound-derived index during controlled hemorrhage and transfusion under PnP and No-PnP conditions. The present analysis used recordings from the same animals and protocol to address a separate question concerning MAP and HPI responses. The HPI data and analyses were not included in the related manuscript. No additional animals or experimental procedures were used.

### 2.2. Ethical Approval, Animals, and Husbandry

The original animal experiment was approved by the Institutional Animal Care and Use Committee of Asan Medical Center (Protocol No. 2025-20-104; approved on 17 April 2025). This study is reported in accordance with the ARRIVE 2.0 guidelines [18]. All procedures were performed in accordance with applicable guidelines and regulations, including the Laboratory Animal Act and the Animal Protection Act. Fourteen Yorkshire pigs (7 females and 7 males), obtained from Kukje Experimental Animal Center (Pocheon, Republic of Korea), with a mean weight of 45.14 ± 4.65 kg, were used. The animals were acclimated for approximately two weeks in the institutional large-animal facility, housed under controlled environmental conditions with standard husbandry and environmental enrichment, and cared for under veterinary supervision. They had free access to water and a standard swine diet during the acclimation period. The protocol specified a humane endpoint of a reduction in body weight of 20% or more relative to the mean body weight of healthy animals of comparable age. Animals meeting this criterion were to be euthanized by intravenous potassium chloride (Potassium Chloride-40 Inj. Daihan; Dai Han Pharm Co., Ltd., Seoul, Republic of Korea) administration under anesthesia. Food was withheld for 12 h before anesthesia, with water available until preanesthetic preparation. This was a terminal, non-recovery experiment.

The experimental unit was the individual animal. The sample size for the parent experiment was planned to obtain at least 12 analyzable pneumoperitoneum datasets. This number was based on an a priori sample size calculation using Fisher’s z transformation to detect a large physiological association under the pneumoperitoneum condition, assuming an expected correlation coefficient of 0.75, a two-sided α of 0.05, and 80% power. Therefore, 14 animals were enrolled to account for possible attrition. The original sample size calculation did not establish power for MAP or HPI condition-by-window interaction tests. The available cohort was used for the present analysis, which was interpreted as an exploratory repeated-measures physiological study. The paired pneumoperitoneum versus no-pneumoperitoneum analyses were restricted to animals with analyzable data under both conditions.

The predefined exclusion criteria were death before completion of the experimental protocol and unavailable or unusable time-synchronized recordings resulting from technical failure.

### 2.3. Anesthesia and Monitoring

General anesthesia was induced with intramuscular zolazepam/tiletamine (Zoletil 50; Virbac, Carros, France) at 5 mg/kg and xylazine (Rompun Inj.; Elanco Animal Health Korea Co., Ltd., Ansan, Republic of Korea) at 2 mg/kg. A 22-gauge auricular venous catheter (BD Angiocath; Becton Dickinson Infusion Therapy Systems Inc., Sandy, UT, USA) was placed. Electrocardiography (ECG) and peripheral oxygen saturation (SpO_2_) were continuously monitored using a multiparameter patient monitor (B125M; GE Medical Systems Information Technologies, Inc., Wauwatosa, WI, USA). After endotracheal intubation, anesthesia was maintained with isoflurane (Ifran Liq.; Hana Pharm Co., Ltd., Hwaseong, Republic of Korea) delivered at a vaporizer setting of 1.5 vol%. Rocuronium (Rocunium Inj.; Hana Pharm Co., Ltd., Hwaseong, Republic of Korea) was administered intravenously as a 25 mg bolus followed by a continuous infusion at 1 mg/kg/h during controlled mechanical ventilation. Neuromuscular block was not assessed during the experiment. The lungs were mechanically ventilated using anesthesia machine (Fabius GS premium; Drägerwerk AG & Co. KGaA, Lübeck, Germany) in volume-controlled mode with a tidal volume of 8 mL/kg, an inspiratory-to-expiratory ratio of 1:2, positive end-expiratory pressure of 5 cm H_2_O, and a fraction of inspired oxygen of 0.4. The respiratory rate was adjusted to maintain end-tidal carbon dioxide between 35 and 45 mmHg. End-tidal carbon dioxide was monitored using anesthesia machine (Fabius GS premium; Drägerwerk AG & Co. KGaA, Lübeck, Germany).

A 20-gauge femoral arterial catheter (BD Angiocath; Becton Dickinson Infusion Therapy Systems Inc., Sandy, UT, USA) was placed for continuous arterial pressure waveform monitoring using an Acumen IQ sensor connected to a HemoSphere monitor (Edwards Lifesciences, Irvine, CA, USA). Arterial blood pressure (systolic, diastolic, and mean) and HPI were recorded from this system. A 16-gauge catheter (BD Angiocath; Becton Dickinson Infusion Therapy Systems Inc., Sandy, UT, USA) was inserted into the right femoral vein for controlled hemorrhage and transfusion. In addition, a pulmonary artery catheter (Swan-Ganz; Edwards Lifesciences, Irvine, CA, USA) was inserted via the right internal jugular vein. Continuous cardiac output (CCO) and systemic vascular resistance were recorded as variables derived from the pulmonary artery catheter (PAC). Transthoracic echocardiography (TTE) was performed using Vivid E90 (GE Vingmed Ultrasound AS, Horten, Norway) at four predefined time points: before hemorrhage, after hemorrhage, before transfusion, and after transfusion. Echocardiographic variables included left ventricular ejection fraction (LVEF) and stroke volume, calculated using Simpson’s method. These variables served as supportive physiological measures of global systolic function and directional stroke volume changes during the hemorrhage–transfusion protocol.

### 2.4. Hemorrhage–Transfusion Protocol

Before initiating the hemorrhage–transfusion protocol, intravenous unfractionated heparin (Heparin Injection Choongwae; JW Pharmaceutical, Gwacheon, Republic of Korea) was administered after induction and instrumentation as a 12,500 IU bolus, followed by 5000 IU every hour for anticoagulation. Blood was manually withdrawn with a syringe through the indwelling right femoral venous catheter in 100 mL increments to a total of 600 mL and subsequently retransfused in 100 mL increments. Throughout withdrawal and retransfusion, the animals were positioned supine with all four limbs secured and remained under general anesthesia, continuous rocuronium infusion, and controlled mechanical ventilation. In a 45 kg pig, a 600 mL hemorrhage corresponds to approximately 20% of the estimated blood volume, based on published blood volume values of approximately 6.5 mL/100 g body weight in swine of comparable size [19], and falls within the range of class II hemorrhage according to Advanced Trauma Life Support criteria [20]. Withdrawn blood was collected and stored in a heparinized blood transfusion bag (Green TF Bag-600; Doowon Meditec Co., Ltd., Gimje, Republic of Korea) until retransfusion. Time stamps were recorded at each 100 mL step to synchronize continuous recordings with protocol intervals.

Because this was a terminal, non-recovery experiment, and the animals remained under general anesthesia until euthanasia, postoperative analgesia was not applicable. No additional anesthetic, analgesic, vasoactive, or experimental drugs were administered during the protocol other than those described above. At the end of the experiment, while the animals were still under deep general anesthesia with mechanical ventilation, euthanasia was performed by intravenous administration of 40 mEq potassium chloride in 20 mL through the venous catheter, in accordance with the IACUC-approved protocol. Death was confirmed by the cessation of the arterial pressure waveform, electrocardiographic activity, and heartbeat.

### 2.5. Study Design and Pneumoperitoneum Conditions

The first three animals formed a pilot cohort studied under PnP conditions only. The subsequent 11 animals were assigned to undergo the protocol under both PnP and No-PnP conditions in a randomized crossover sequence, with condition order determined using a computer-based order generator using the built-in RAND() function in Microsoft Excel for Microsoft 365 (version 2610, build 16.0.20527.20088; Microsoft Corporation, Redmond, WA, USA). Randomization and outcome measurements were performed by the investigator conducting the experiment, who was aware of the condition assignments because pneumoperitoneum was evident from abdominal distension. Data analysis was performed by a separate investigator who was not involved in the experimental procedures and was blinded to the PnP and No-PnP condition assignments.

For the PnP condition, CO_2_ was insufflated through a single umbilical port and maintained at an intra-abdominal pressure of 15 mmHg using a laparoscopic insufflator (PneumoSure XL; Stryker Endoscopy, San Jose, CA, USA). The selected pressure is consistent with a previous porcine study of CO_2_ pneumoperitoneum [21]. After reaching the target pressure, physiological variables were allowed to stabilize for 10 min before initiating the hemorrhage–transfusion protocol. One hemorrhage–transfusion set was defined as the completion of the hemorrhage and transfusion sequence under a given condition (PnP or No-PnP).

### 2.6. MAP and HPI Outcomes

The outcomes of interest were MAP and HPI. MAP was measured in mmHg, while HPI was expressed as a dimensionless score ranging from 0 to 100, generated by the monitoring system based on the arterial pressure waveform [6]. Both MAP and HPI were assessed as physiological responses across protocol windows during hemorrhage and transfusion. Other hemodynamic variables were summarized as supportive physiological measurements.

### 2.7. Data Acquisition and Extraction

Hemodynamic waveforms and monitor-derived numeric variables—including systolic, diastolic, and mean arterial pressures; heart rate; HPI; continuous cardiac output; and systemic vascular resistance—were recorded continuously and time-synchronized using SignalTAB (version 1.0.0; Signal House Co., Ltd., Seoul, Republic of Korea), an Android-based biosignal acquisition application [22]. Event markers were logged at the start and end of hemorrhage, the start and end of transfusion, and at each cumulative 100 mL increment (100–600 mL) during both the hemorrhage and transfusion phases.

For offline analysis, data were segmented into prespecified protocol windows within each phase, including a 60 s baseline window (pre-hemorrhage or pre-transfusion), six cumulative volume intervals defined by successive 100 mL increments (0–100 mL through 500–600 mL), and a 60 s post-phase window (post-hemorrhage or post-transfusion). Window-level values were calculated as the arithmetic mean of all available samples after preprocessing.

Echocardiographic variables were analyzed at four predefined time points and summarized as paired, phase-specific measurements: pre- and post-hemorrhage for the hemorrhage phase, and pre- and post-transfusion for the transfusion phase. Because echocardiography was not performed at every 100 mL interval, interval-specific echocardiographic values were unavailable.

### 2.8. Statistical Analysis

Analyses were performed separately for the hemorrhage and transfusion phases. For each outcome, a linear mixed-effects model was fitted with condition (PnP vs. No-PnP), protocol window, and their interaction as fixed effects, and animal as a random intercept to account for within-animal correlation due to repeated measurements. The protocol window was treated as a categorical variable, with the phase-specific baseline window serving as the reference level (Pre-hemorrhage for the hemorrhage phase and Pre-transfusion for the transfusion phase). This model was used to assess the effects of condition, protocol window, and their interaction, as well as to estimate model-based trajectories across the protocol windows.

A prespecified secondary analysis evaluated the within-animal PnP effect, defined as Δ = PnP − No-PnP within the same animal. This analysis was restricted to animals with paired observations under both conditions. Linear mixed-effects models, with protocol window as a fixed effect and animal as a random intercept, were used to assess the effect of the window on Δ, thereby isolating the effect of PnP while reducing between-animal variability in baseline physiology.

For descriptive reporting, data are presented as means ± standard deviations by condition and protocol window. Post hoc comparisons were conducted using model-based contrasts. For continuously recorded hemodynamic variables, seven comparisons against the phase-specific baseline were performed per variable and condition. Seven comparisons against the phase-specific baseline Δ were also performed per outcome and phase. Bonferroni correction was applied only to the eight interval-specific PnP-versus-No-PnP contrasts per outcome and phase. All other post hoc comparisons were unadjusted and considered exploratory. Model-based trajectories were visualized using estimated marginal means with 95% confidence intervals derived from the fitted models. For linear mixed-effects models, model convergence was assessed. Residual-versus-fitted plots and quantile–quantile plots of residuals were visually inspected to evaluate homoscedasticity and approximate residual normality. All tests were two-sided. Statistical significance was defined as *p* < 0.05.

Analyses were performed using Python (version 3.10), with NumPy (version 1.26.4) and pandas (version 2.3.3) for numerical computation and data handling, statsmodels (version 0.14.6) for linear mixed-effects modeling, and SciPy (version 1.13.1) for statistical testing, including the Wilcoxon signed-rank test.

Echocardiographic variables were analyzed separately for the PnP and No-PnP conditions because measurements were obtained at paired, phase-specific time points rather than across protocol windows. Within each condition, pre- and post-phase measurements during hemorrhage and transfusion were compared using the two-sided Wilcoxon signed-rank test on paired, analyzable data due to the limited sample size. Echocardiographic data are presented as mean ± standard deviation to maintain consistency with the main tables.

## 3. Results

### 3.1. Study Animals and Analyzed Datasets

Fourteen animals were enrolled in the experiment. Three pilot animals underwent the protocol under PnP conditions only, and 11 animals were assigned to a randomized crossover sequence involving both PnP and No-PnP conditions. Of the crossover animals, one died during anesthetic induction and one was excluded because recordings were unavailable owing to technical failure. The remaining nine crossover animals each contributed one set under each condition. Thus, 12 animals contributed 21 analyzed hemorrhage–transfusion sets: 12 PnP sets (three pilot and nine crossover sets) and nine No-PnP sets. Each set comprised a hemorrhage–transfusion sequence under one condition.

### 3.2. MAP and HPI During Hemorrhage and Transfusion

The number of animals with analyzable observations varied depending on the outcome and protocol window. MAP data were available from 12 animals under the PnP protocol, and from 8 to 9 animals under the No-PnP protocol. HPI data were available from 11 animals under PnP and 8 to 9 animals under No-PnP (Table 1 and Table 2).

During hemorrhage, MAP decreased and HPI showed an overall increase in both conditions (all *p* < 0.001; Table 1). MAP decreased from 77.4 ± 11.0 mmHg to 60.5 ± 12.0 mmHg under PnP and from 73.7 ± 13.3 mmHg to 61.4 ± 11.5 mmHg under No-PnP. HPI increased from 50.3 ± 33.3 to 72.3 ± 40.3 under PnP and from 47.1 ± 35.9 to 75.5 ± 29.2 under No-PnP. Both systolic and diastolic arterial pressures, as well as continuous cardiac output, decreased in both conditions (all *p* < 0.001). Heart rate increased significantly under PnP (*p* < 0.001) but not under No-PnP (*p* = 0.073). TTE variables showed that Simpson-derived stroke volume decreased after hemorrhage under both PnP and No-PnP conditions, whereas LVEF did not change significantly (Table 1).

During transfusion, MAP increased and HPI showed an overall decrease in both conditions (all *p* < 0.001; Table 2). MAP increased from 66.4 ± 12.4 mmHg to 83.1 ± 10.8 mmHg under PnP and from 64.7 ± 10.2 mmHg to 79.1 ± 12.8 mmHg under No-PnP. HPI decreased from 68.1 ± 39.9 to 32.6 ± 27.9 under PnP and from 72.9 ± 34.4 to 43.8 ± 30.5 under No-PnP. Both systolic and diastolic arterial pressures, as well as systemic vascular resistance, increased in both conditions (all *p* < 0.001). Heart rate decreased under PnP (*p* < 0.001) but remained relatively stable under No-PnP (*p* = 0.912). TTE variables showed that Simpson-derived stroke volume increased after transfusion under both PnP and No-PnP conditions, whereas LVEF did not change significantly (Table 2).

### 3.3. PnP-Related Patterns of MAP and HPI Change

**Figure 1:**
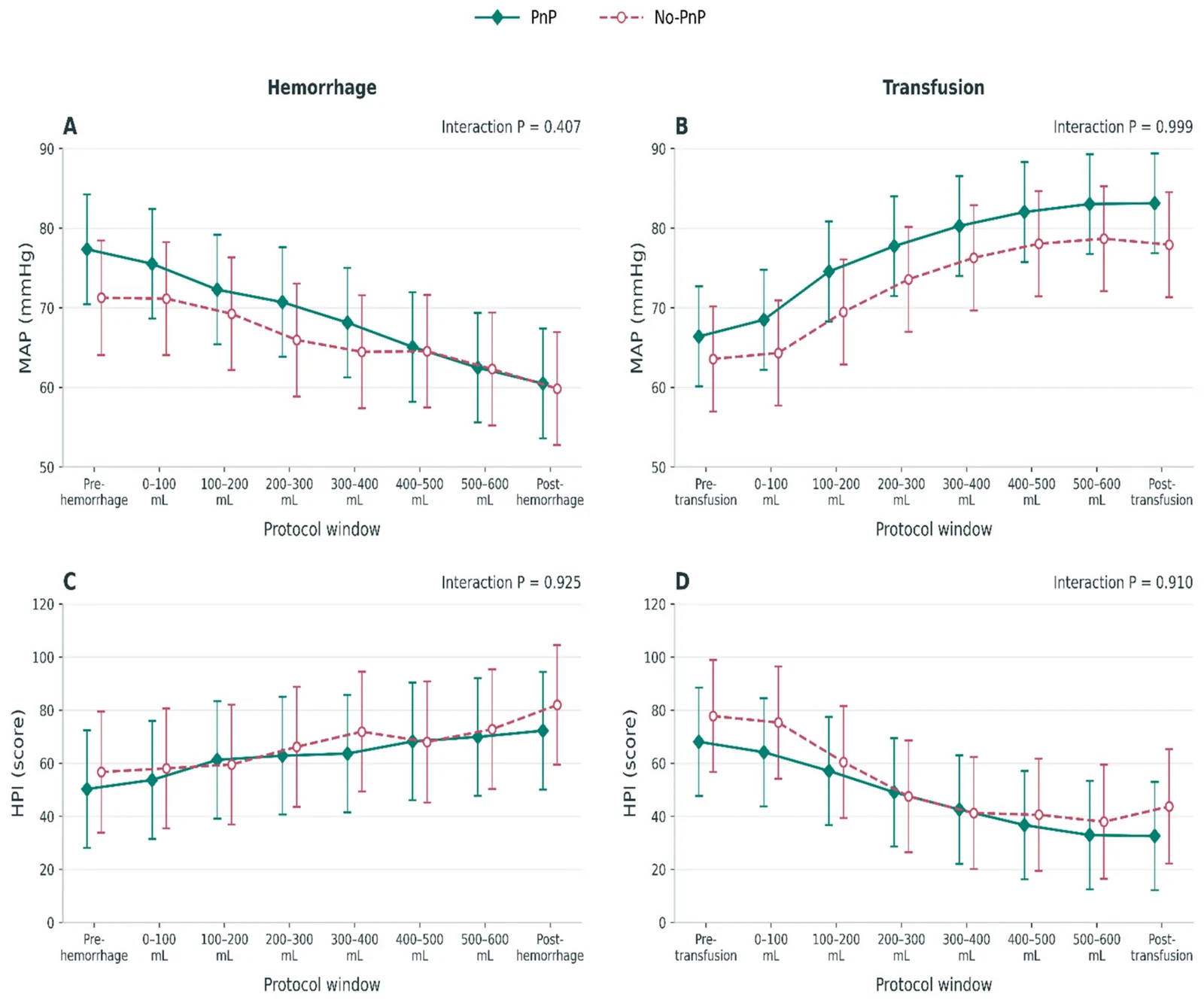
Model-estimated trajectories of MAP and HPI during stepwise hemorrhage and transfusion intervals under pneumoperitoneum (PnP) and no pneumoperitoneum (No-PnP) conditions. (**A**) MAP during hemorrhage; (**B**) MAP during transfusion; (**C**) HPI during hemorrhage; (**D**) HPI during transfusion.

No statistically significant condition-by-window interaction was detected for MAP or HPI during hemorrhage (*p* = 0.407 and *p* = 0.925, respectively; Figure 1). Similarly, no significant interaction was detected during transfusion (MAP, *p* = 0.999; HPI, *p* = 0.910). Thus, the models did not reveal statistically significant differences in the patterns of protocol-window changes between the PnP and No-PnP conditions.

Filled teal diamonds with solid connecting lines indicate PnP, and open rose circles with dashed connecting lines indicate No-PnP. Points represent estimated marginal means from linear mixed-effects models including condition, protocol window, and their interaction as fixed effects, with animal as a random intercept. Vertical error bars indicate 95% confidence intervals. The two conditions are slightly offset horizontally within each protocol window for legibility; the offsets do not represent different measurement times. Displayed *p* values refer to the condition-by-window interaction. Eight interval-specific PnP versus No-PnP contrasts were Bonferroni-adjusted within each phase-by-outcome panel; none reached an adjusted *p* < 0.05. MAP, mean arterial pressure; HPI, hypotension prediction index; PnP, pneumoperitoneum; No-PnP, no pneumoperitoneum.

### 3.4. Interval-Specific Within-Animal Differences Between Conditions

**Figure 2:**
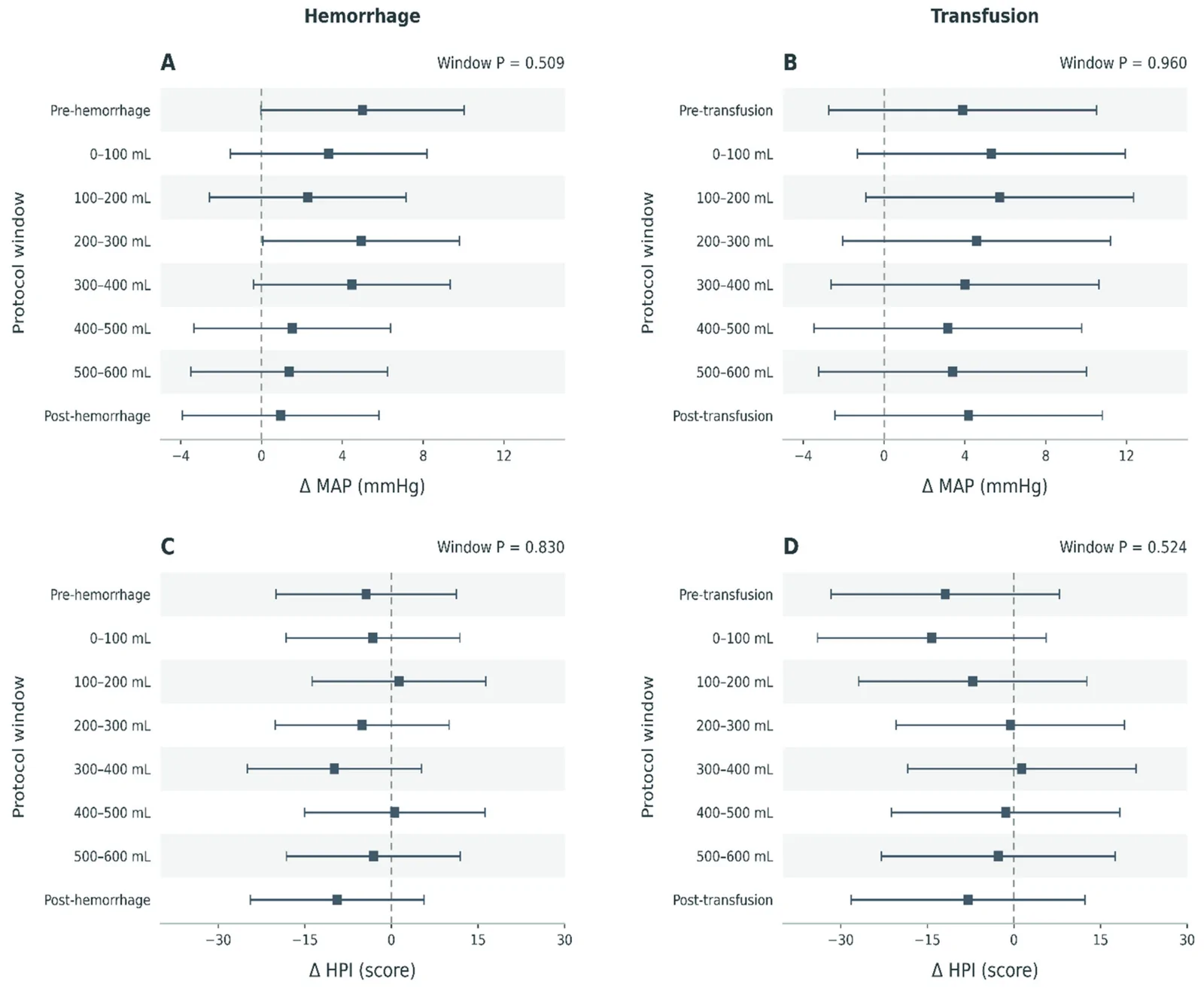
Interval-specific within-animal differences between conditions (Δ = PnP − No-PnP) for MAP and HPI during hemorrhage and transfusion. (**A**) MAP during hemorrhage; (**B**) MAP during transfusion; (**C**) HPI during hemorrhage; (**D**) HPI during transfusion.

Within-animal MAP differences were numerically positive throughout both phases but did not change significantly across protocol windows during hemorrhage (*p* = 0.509) or transfusion (*p* = 0.960). Within-animal HPI differences fluctuated around predominantly negative values, with no statistically significant effect of protocol window during hemorrhage (*p* = 0.830) or transfusion (*p* = 0.524; Figure 2).

Forest plots show the within-animal difference, Δ = PnP − No-PnP, for each protocol window. Panels A and B show MAP during hemorrhage and transfusion, respectively; panels C and D show HPI during the corresponding phases. Slate squares represent model-based estimates, and horizontal error bars indicate 95% confidence intervals from linear mixed-effects models including protocol window as a fixed effect and animal as a random intercept. The dashed vertical line at zero indicates no difference between conditions; positive values indicate higher values under PnP. Analysis was restricted to paired observations (*n* = 8–9 per window, depending on the outcome and protocol window). Displayed *p* values refer to the overall protocol-window effect on Δ. Seven contrasts against the phase-specific baseline Δ were evaluated without multiplicity adjustment within each phase-by-outcome panel; none reached *p* < 0.05. MAP, mean arterial pressure; HPI, hypotension prediction index; PnP, pneumoperitoneum; No-PnP, no pneumoperitoneum.

## 4. Discussion

In this porcine model of controlled, stepwise hemorrhage and retransfusion, PnP did not significantly alter the patterns of MAP and HPI change. In both conditions, MAP decreased and HPI increased overall during hemorrhage, with the opposite pattern observed during transfusion. Neither outcome showed a significant condition-by-window interaction, and within-animal differences between PnP and No-PnP did not vary significantly across protocol windows in either phase. These findings do not establish equivalence between the conditions.

The accompanying PAC and TTE findings help contextualize these trajectories. During hemorrhage, both CCO and Simpson-derived stroke volume decreased under both conditions, consistent with the expected reduction in forward flow. During retransfusion, Simpson-derived stroke volume increased under both conditions, whereas LVEF did not change significantly. These findings are consistent with reversible, load-dependent changes during the hemorrhage–transfusion protocol, although preserved LVEF alone does not confirm unchanged intrinsic contractility. Systemic vascular resistance also increased during transfusion, indicating that the recovery in MAP occurred alongside changes in both stroke volume and vascular resistance. Previous clinical studies have shown that PnP can increase arterial pressure and systemic vascular resistance despite a reduction in cardiac output [9,11]. Changes in ventricular loading and neurohumoral responses may contribute to these effects [10,11]. Therefore, MAP and HPI responses should be interpreted in the context of concurrent changes in forward flow, ventricular loading, and vascular tone.

Our observations are consistent with previous experimental evaluations of HPI during hemodynamic disturbances. In a porcine model involving blood withdrawal of up to 10% of the estimated blood volume, increases in HPI and decreases in MAP were accompanied by impaired gastrointestinal microcirculation [16]. HPI increased during esmolol-induced negative inotropy in another porcine study [17]. Collectively, these findings suggest that HPI responses are not specific to blood volume depletion. The present study extends this physiological evaluation by comparing responses during the same hemorrhage–transfusion protocol under PnP and No-PnP conditions. The overall decline in HPI during retransfusion is consistent with a response to the restoration of circulating volume but does not elucidate the underlying mechanism or establish a threshold for fluid treatment. The present analysis used data from the same animal experiment as the evaluation of pulse pressure variation, stroke volume variation, and heart sound-derived index and therefore does not represent an independent experimental replication. Its findings are specific to the shared hemorrhage–transfusion protocol and inherit the same limitations related to anesthesia, ventilation, PnP pressure, and the crossover design. The present contribution is the characterization of MAP and HPI responses within that experimental setting.

This study has several strengths. We used a controlled, stepwise hemorrhage–retransfusion model that enables systematic evaluation of bidirectional changes in circulating blood volume. Blood withdrawal and replacement were conducted in 100 mL increments, with standardized PnP pressure, anesthetic, and ventilatory protocols. The crossover design allowed for within-animal comparisons, minimizing the influence of baseline physiological differences. Repeated measurements were analyzed using mixed-effects models, and the paired analysis offered a complementary assessment limited to animals with observations under both conditions.

Several limitations should be considered. This study was an exploratory secondary analysis, and the original sample size calculation was not designed to detect condition-by-window interaction effects for MAP or HPI. The small cohort size, substantial variability in HPI, and incomplete paired observations limited the precision of the results; therefore, nonsignificant interactions do not rule out clinically relevant differences between conditions. The main analysis included three pilot animals studied only under PnP, and period and carryover effects were not explicitly modeled despite the randomized condition order in the crossover cohort. Neuromuscular block was not assessed, limiting verification of its depth and consistency across conditions. Averaging measurements within volume-defined protocol windows may obscure transient changes and the temporal relationship between HPI elevation and subsequent hypotension. Prediction lead time, discrimination, and the incremental predictive value of HPI over contemporaneous MAP were not assessed; thus, these findings do not establish its predictive performance during PnP. Finally, the experiment was conducted in anesthetized pigs at a single PnP pressure of 15 mmHg under standardized conditions, limiting generalizability to the varied surgical positions, cardiovascular comorbidities, and vasoactive interventions encountered in clinical practice. HPI calibration and predictive performance in pigs were also not established in this study, and extrapolation to human laparoscopic surgery requires further validation. Future studies should evaluate MAP and HPI responses in larger cohorts across different PnP pressures and surgical settings. Event-based analyses using continuous recordings are needed to determine prediction lead time and whether HPI provides additional predictive information beyond contemporaneous MAP during PnP. Clinical studies should then assess whether this information can guide hemodynamic interventions and reduce intraoperative hypotension during laparoscopic surgery.

## 5. Conclusions

In this porcine model of controlled, stepwise hemorrhage and retransfusion, no significant PnP-related differences in the patterns of MAP and HPI change were detected. MAP decreased and HPI increased during hemorrhage, with the opposite pattern occurring during retransfusion under both conditions. Further studies are needed to determine whether HPI provides additional predictive information beyond MAP during PnP and whether this information can enhance hemodynamic management during laparoscopic surgery.

## Figures and Tables

**Table 1:** Measured Parameters by Condition during the Hemorrhage Phase.

Conditions	Parameters	Cumulative Hemorrhage Volume								*p*-Value
		Pre-Hemorrhage	0–100 mL	100–200 mL	200–300 mL	300–400 mL	400–500 mL	500–600 mL	Post-Hemorrhage	
PnP	**MAP and HPI**									
(*n* = 12)	MAP, mmHg	77.4 ± 11.0	75.5 ± 10.7	72.3 ± 11.9 *	70.7 ± 14.0 ‡	68.1 ± 14.5 §	65.1 ± 13.6 §	62.5 ± 12.7 §	60.5 ± 12.0 §	<0.001
	HPI	50.3 ± 33.3	53.7 ± 32.7	61.3 ± 38.7	62.9 ± 41.7	63.6 ± 42.6	68.2 ± 43.3 *	69.9 ± 42.4 *	72.3 ± 40.3 †	<0.001
	**Other Vital Signs**									
	HR, beats/min	121.0 ± 33.8	121.1 ± 34.2	123.8 ± 35.8	127.4 ± 37.1	131.3 ± 36.5 †	133.6 ± 33.9 †	136.7 ± 34.9 §	140.2 ± 35.6 §	<0.001
	SBP, mmHg	100.7 ± 13.2	98.4 ± 13.6	93.8 ± 15.9 †	90.8 ± 18.3 §	87.5 ± 19.3 §	82.5 ± 18.1 §	78.4 ± 16.4 §	75.8 ± 16.0 §	<0.001
	DBP, mmHg	58.9 ± 10.0	57.7 ± 9.4	55.7 ± 9.8	54.7 ± 10.9 *	52.9 ± 10.5 ‡	50.4 ± 9.2 §	48.8 ± 8.9 §	47.7 ± 8.4 §	<0.001
	**PAC variables**									
	Analyzed, *n*	11	11	11	11	11	11	11	11	
	CCO, L/min	7.4 ± 1.4	7.4 ± 1.5	7.3 ± 1.5	7.2 ± 1.5	7.0 ± 1.5	6.8 ± 1.6 *	6.7 ± 1.6 †	6.5 ± 1.7 ‡	<0.001
	SVR, dyn·s/cm^5^	761.7 ± 199.6	741.7 ± 175.1	716.1 ± 160.8	717.2 ± 172.7	714.2 ± 178.1	702.2 ± 174.0 *	699.3 ± 173.3 *	690.4 ± 148.7 †	0.087
	**TTE variables**									
	LVEF, %	64.6 ± 3.7	—	—	—	—	—	—	67.0 ± 4.9	0.064
	SV, mL	33.8 ± 9.6	—	—	—	—	—	—	26.6 ± 9.3	<0.001
No-PnP	**MAP and HPI**									
(*n* = 9)	MAP, mmHg	73.7 ± 13.3	72.7 ± 13.3	70.8 ± 12.3	67.5 ± 11.6	66.1 ± 12.4 *	66.1 ± 12.6 *	63.9 ± 12.2 †	61.4 ± 11.5 §	<0.001
	HPI	47.1 ± 35.9	51.6 ± 37.5	53.0 ± 37.8	59.7 ± 34.1	65.4 ± 37.4	68.1 ± 32.8	66.3 ± 31.3	75.5 ± 29.2 †	<0.001
	**Other Vital Signs**									
	HR, beats/min	140.5 ± 28.1	139.8 ± 27.1	141.9 ± 28.0	144.6 ± 30.4	147.3 ± 32.0	150.7 ± 33.1 *	149.7 ± 29.0 *	146.7 ± 27.1	0.073
	SBP, mmHg	93.9 ± 16.3	94.1 ± 17.5	91.5 ± 15.9	86.8 ± 14.4 *	84.1 ± 16.0 ‡	83.9 ± 16.9 ‡	81.4 ± 16.4 §	79.4 ± 16.6 §	<0.001
	DBP, mmHg	53.0 ± 10.0	53.2 ± 10.1	51.7 ± 9.9	49.4 ± 9.8	48.8 ± 10.1 *	49.2 ± 9.8	47.7 ± 9.1 †	45.4 ± 7.4 ‡	<0.001
	**PAC variables**									
	Analyzed, *n*	8	9	9	9	9	9	9	9	
	CCO, L/min	8.0 ± 1.3	8.0 ± 1.3	8.0 ± 1.4	8.0 ± 1.5	7.9 ± 1.6	7.8 ± 1.6	7.6 ± 1.6	7.3 ± 1.6 *	<0.001
	SVR, dyn·s/cm^5^	692.2 ± 133.0	689.3 ± 133.4	678.8 ± 144.5	661.6 ± 165.4	653.7 ± 168.1	667.5 ± 179.4	669.2 ± 203.8	657.2 ± 159.8	0.593
	**TTE variables**									
	LVEF, %	65.7 ± 4.4	—	—	—	—	—	—	67.5 ± 4.1	0.250
	SV, mL	37.0 ± 13.1	—	—	—	—	—	—	30.7 ± 11.6	0.004

Data are presented as mean ± SD. For monitor-derived variables, *n* = 12 under PnP and *n* = 9 under No-PnP unless otherwise indicated. HPI had *n* = 11 throughout under PnP. Under No-PnP, *n* = 8 for HR, MAP, and HPI at pre-hemorrhage and for HPI at 400–500 mL. For CCO and SVR, sample sizes are shown in the “Analyzed, *n*” row. TTE variables were measured only before and after hemorrhage; interval-specific values are unavailable. *p* values indicate within-condition protocol-window effects for monitor-derived variables and two-sided Wilcoxon signed-rank comparisons of pre- and post-hemorrhage values for TTE variables. Symbols indicate adjusted model-based contrasts versus pre-hemorrhage (* *p* < 0.05, † *p* < 0.01, ‡ *p* < 0.001, § *p* < 0.0001). CCO, continuous cardiac output; DBP, diastolic blood pressure; HPI, hypotension prediction index; HR, heart rate; LVEF, left ventricular ejection fraction; MAP, mean arterial pressure; No-PnP, no pneumoperitoneum; PAC, pulmonary artery catheter; PnP, pneumoperitoneum; SBP, systolic blood pressure; SD, standard deviation; SV, stroke volume; SVR, systemic vascular resistance; TTE, transthoracic echocardiography.

**Table 2:** Measured Parameters by Condition during the Transfusion Phase.

Conditions	Parameters	Cumulative Transfusion Volume								*p*-Value
		Pre-Transfusion	0–100 mL	100–200 mL	200–300 mL	300–400 mL	400–500 mL	500–600 mL	Post-Transfusion	
PnP	**MAP and HPI**									
(*n* = 12)	MAP, mmHg	66.4 ± 12.4	68.5 ± 13.0	74.6 ± 12.5 ‡	77.7 ± 11.1 §	80.3 ± 10.6 §	82.0 ± 10.3 §	83.0 ± 10.8 §	83.1 ± 10.8 §	<0.001
	HPI	68.1 ± 39.9	64.2 ± 42.2	57.1 ± 41.2	49.1 ± 36.5	42.6 ± 33.7 †	36.7 ± 29.9 ‡	32.9 ± 28.1 §	32.6 ± 27.9 §	<0.001
	**Other Vital Signs**									
	HR, beats/min	139.8 ± 36.9	139.5 ± 36.2	136.4 ± 35.5	133.6 ± 35.8	129.7 ± 35.5 *	128.2 ± 35.1 *	127.1 ± 34.8 †	126.0 ± 34.2 †	<0.001
	SBP, mmHg	82.9 ± 16.0	85.6 ± 16.6	93.9 ± 15.6 ‡	98.4 ± 14.0 §	102.0 ± 13.5 §	104.8 ± 12.8 §	105.5 ± 13.3 §	105.8 ± 13.0 §	<0.001
	DBP, mmHg	52.0 ± 9.6	53.7 ± 10.1	57.9 ± 10.4 †	60.1 ± 9.5 ‡	61.8 ± 9.0 §	62.9 ± 9.3 §	63.5 ± 9.4 §	63.5 ± 9.4 §	<0.001
	**PAC variables**									
	Analyzed, *n*	11	11	11	11	10	10	10	5	
	CCO, L/min	6.0 ± 1.8	5.9 ± 1.8	5.9 ± 1.7	5.9 ± 1.7	5.9 ± 1.8	5.9 ± 1.8	5.9 ± 1.8	6.2 ± 1.6	<0.001
	SVR, dyn·s/cm^5^	828.6 ± 170.4	879.8 ± 192.5	956.3 ± 225.4 †	998.5 ± 236.1 §	1036.8 ± 283.6 §	1056.7 ± 311.7 §	1022.9 ± 311.7 §	967.9 ± 222.8 §	<0.001
	**TTE variables**									
	LVEF, %	67.0 ± 4.9	—	—	—	—	—	—	66.2 ± 4.3	0.424
	SV, mL	26.6 ± 9.3	—	—	—	—	—	—	35.6 ± 10.9	<0.001
No-PnP	**MAP and HPI**									
(*n* = 9)	MAP, mmHg	64.7 ± 10.2	65.5 ± 10.5	70.6 ± 11.9 *	74.7 ± 11.2 ‡	77.4 ± 11.2 §	79.2 ± 12.3 §	79.8 ± 12.3 §	79.1 ± 12.8 §	<0.001
	HPI	72.9 ± 34.4	70.4 ± 35.3	55.5 ± 34.6	42.6 ± 35.0 †	36.3 ± 33.1 ‡	35.7 ± 33.0 ‡	38.1 ± 31.2 §	43.8 ± 30.5 ‡	<0.001
	**Other Vital Signs**									
	HR, beats/min	147.8 ± 27.8	147.2 ± 28.1	147.8 ± 28.4	149.4 ± 30.0	148.6 ± 30.4	146.8 ± 29.7	145.6 ± 29.3	142.0 ± 28.9	0.912
	SBP, mmHg	82.9 ± 13.4	83.7 ± 12.8	90.1 ± 13.4 *	96.0 ± 13.0 ‡	99.0 ± 12.4 §	101.5 ± 14.1 §	102.3 ± 14.0 §	101.2 ± 14.7 §	<0.001
	DBP, mmHg	47.7 ± 7.7	48.2 ± 8.3	52.0 ± 9.7	55.3 ± 9.1 †	57.1 ± 9.5 ‡	58.9 ± 10.6 §	59.5 ± 10.5 §	58.9 ± 11.0 §	<0.001
	**PAC variables**									
	Analyzed, *n*	9	9	9	9	9	9	8	6	
	CCO, L/min	6.8 ± 1.6	6.7 ± 1.6	6.6 ± 1.6	6.6 ± 1.6	6.6 ± 1.6	6.6 ± 1.6	6.5 ± 1.7	6.8 ± 1.9 *	<0.001
	SVR, dyn·s/cm^5^	756.8 ± 205.9	782.6 ± 223.7	845.3 ± 255.2	893.9 ± 262.5 †	924.6 ± 271.7 ‡	957.3 ± 289.3 ‡	954.1 ± 270.4 ‡	914.6 ± 302.2 †	<0.001
	**TTE variables**									
	LVEF, %	67.5 ± 4.1	—	—	—	—	—	—	65.9 ± 3.5	0.426
	SV, mL	30.7 ± 11.6	—	—	—	—	—	—	36.8 ± 13.3	0.004

Data are presented as mean ± SD. For monitor-derived variables, *n* = 12 under PnP and *n* = 9 under No-PnP unless otherwise indicated. Under PnP, HPI had *n* = 11 throughout, and SBP had *n* = 11 at 400–500 mL. Under No-PnP, HPI had *n* = 8 at 500–600 mL and post-transfusion. The “Analyzed, *n*” row applies to CCO and SVR, except that No-PnP SVR had *n* = 8 at 400–500 mL. TTE variables were measured only before and after transfusion; interval-specific values are unavailable. *p* values indicate within-condition protocol-window effects for monitor-derived variables and two-sided Wilcoxon signed-rank comparisons of pre- and post-transfusion values for TTE variables. Symbols indicate adjusted model-based contrasts versus pre-transfusion (* *p* < 0.05, † *p* < 0.01, ‡ *p* < 0.001, § *p* < 0.0001). CCO, continuous cardiac output; DBP, diastolic blood pressure; HPI, hypotension prediction index; HR, heart rate; LVEF, left ventricular ejection fraction; MAP, mean arterial pressure; No-PnP, no pneumoperitoneum; PAC, pulmonary artery catheter; PnP, pneumoperitoneum; SBP, systolic blood pressure; SD, standard deviation; SV, stroke volume; SVR, systemic vascular resistance; TTE, transthoracic echocardiography.

## Data Availability

The datasets presented in this article are not publicly available due to institutional data-sharing restrictions. Requests for access to the datasets should be directed to the corresponding author.
